# An Irregularity Measurement Based Cardiac Status Recognition Using Support Vector Machine

**DOI:** 10.1155/2015/327534

**Published:** 2015-10-27

**Authors:** Poulami Banerjee, Ashok Mondal

**Affiliations:** Department of Electronics and Communication Engineering, Maulana Azad National Institute of Technology, Bhopal 462003, India

## Abstract

An automated robust feature extraction technique is proposed in this paper based on inherent structural distribution of heart sound to analyze the phonocardiogram signal in presence of environmental noise and interference of lung sound signal. The structural complexity of the heart sound signal is estimated in terms of sample entropy using a nonlinear signal processing framework. The effectiveness of the feature is evaluated using a support vector machine under two different circumstances which include Gaussian noise and pulmonary perturbation. The analysis framework has been executed on a composite data set of 60 healthy and 60 pathological individuals for different SNR levels (−5 to 10 dB) and the performance accuracy is close to that of the clean signal. In addition, a comparative study has been done with conventional approaches which includes waveform analysis, spectral domain inspection, and spectrogram evaluation. The experimental results show that sample entropy based classification method gives an accuracy of 96.67% for clean data and 91.66% for noisy data of SNR 10 dB. The result suggests that the proposed method performs significantly well over the visual and audio test.

## 1. Introduction

Heart sounds are produced due to blood circulation through the heart valves. Recorded phonocardiogram (PCG) signal from a normal subject comprises four sporadic parts, of which two fundamental components are S1 and S2. During ventricular contraction, closure of the mitral and tricuspid valves results in the occurrence of the first heart sound S1. S2, the second heart sound, is produced due to closing of pulmonary and aortic valves during ventricular relaxation [1]. The other two constituents of the heart sound signal with low amplitude and low frequency components are S3 and S4. The third and the forth components and the heart murmurs occur in the systolic and diastolic interval. Murmurs are produced due to several reasons like abnormal blood flow through the valves, defects in opening or closing of the valves, and blockage inside the cardiovascular system [2]. The heart murmurs play a crucial role in detection of heart diseases.

The pathological status of the heart can be diagnosed at an elementary level by using auscultation. This procedure has been used since the invention of stethoscope by a French doctor Laennec in 1816 [3]. Cardiac auscultatory proficiency of physicians plays an important role in diagnosis of heart disease. Moreover, the process of auscultation is effected by environmental noise and also the interference of the lung sound with the heart sound.

To overcome the limitation of the auscultation process, nowadays cardiologists have easy access to various invasive and noninvasive ways for diagnosis of heart diseases. The invasive techniques include echocardiograph and angiography [2]. With the development of technologies in the field of digital signal processing, several tests are being conducted to detect the dysfunctioning of heart like echocardiogram, cardiac computed tomography, and so forth [4]. Amongst these noninvasive methods one of the most widely used is echocardiogram (ECG). However, the performances of these photographic techniques depend on the physician skill, knowledge, and experience. Furthermore, this is a time consuming process and its effectiveness degrades under noisy condition. A patient with heart disorders resulting from abnormalities in mechanical functioning are not always correctly identifiable by echocardiogram [5]. CT scan is also time consuming and can even be harmful for repetitive injection of X-ray into the body.

The drawbacks of the auscultation process and photographic techniques motivate the researchers to develop new methods for better interpretation of cardiac sounds. Several investigations have been done to analyze heart sound signal based on machine learning and pattern recognition approaches [2, 6–14]. Most of these techniques consist of several stages such as data acquisition phase, preprocessing stage, feature extraction phase, and decision making operation. In the first intermediate stage, that is, preprocessing, irrelevant information like noise and artifacts removal operation is performed. The second intermediate stage involved with pattern recognition task is based on temporal, spectral, tempospectral, and statistical domain features. However, the multilayer based detection processes are time consuming due to multiple operations and their performance depends on several factors associated with different stages. Hence, these approaches are not suitable for real-time applications.

From the perception of clinicians, there is a need to analyze heart sound signal in hospital environment for a small period of time. In this paper, a new robust feature extraction technique has been proposed to analyze the phonocardiogram signal in noisy environment. This method includes two stages. In this approach, the preprocessing stage is excluded in order to speed up the decision making process without any tradeoff with accuracy. First, the relevant inherent attributes of the signals are extracted in terms of irregularity measurement parameter or sample entropy (SampEnt). After feature extraction, output of this intermediate stage is fed to the input of the classifier for decision making. The rest of the chapter is organized as follows.  Section 2 illustrates the concept of support vector machine (SVM) and Section 3 gives a description about the data acquisition system and database. The various stages of cardiac status detection system are discussed in Section 4. This is followed by the experimental results and discussion in Section 5. Finally conclusion is given in Section 6.

## 2. Theoretical Background

### 2.1. Support Vector Machine (SVM)

The support vector machine (SVM) network was proposed by Cortes and Vapnik in 1995 as an alternative tool of multilayer feedforward neural network [15]. SVMs are used to solve the classification and regression problems. The SVMs classify different patterns through the two steps: (1) first the training data are mapped to a feature space of high dimension using a nonlinear kernel function and (2) after that an optimal hyperplane is constructed using the method of Lagrange multipliers in order to separate the individual classes [16]. The hyperplane is used to distinguish two linearly separable classes as given by

(1) $$\begin{array}{c} d_{j}\left(\;\omega^{T}f\left(\;y_{j}\;\right)+b\;\right)\geq 1\;\text{for }j=1,2,\ldots ,L, \end{array}$$

where *y*_*j*_ ∈ **R** ^*n*^ is *j*th input pattern and *d*_*j*_ ∈ {−1,1} is the corresponding output pattern or target for a training data set {*y*_*j*_, *d*_*j*_}_*j*=1_^*L*^. *f*(·) is a nonlinear mapping function.

The decision surface of (1) is modified by introducing a nonnegative slack variable *ξ* in order to separate two nonlinearly separable classes and represented by

(2) $$\begin{array}{c} d_{j}\left[\;\omega^{T}f\left(\;y_{j}\;\right)+b\;\right]\geq 1-\xi_{j}\;\text{for }j=1,2,\ldots ,L. \end{array}$$

An optimal hyperplane can be obtained by minimizing the function *F*(*ω*, *ξ*) with respect to *ω* and *ξ*_*j*_ and it is expressed by

(3) $$\begin{array}{c} F\left(\;\omega ,\xi \;\right)=\frac{1}{2}\omega^{T}\omega +\Gamma \overset{L}{\underset{j=1}{\sum }}\xi_{j}, \end{array}$$

where Γ is the reciprocal of a regularization parameter and it controls the tradeoff between complexity of the machine and the number of nonseparable points [17].

To construct a decision function *ϕ*(*y*) (equation (4)) for a SVM classifier, it is required to maximize the objective function *Q*_*f*_(*α*) with respect to Lagrange multipliers {*α*_*j*_}_*j*=1_^*L*^, subject to the two constraints as expressed by (6):

(4) ϕy=sign⁡∑j=1LαjdjKfy,yj+b,

(5) Qfα=∑j=1Lαj−12∑j=1L ∑i=1LαjαiKfyj,yidjdi

subject to

(6) $$\begin{array}{c} \overset{L}{\underset{j=1}{\sum }}d_{j}\alpha_{j}=0,\;0\leq \alpha_{j}\leq \Gamma ,\;\;j=1,2,\ldots ,L. \end{array}$$

The kernel function *K*_*f*_(*y*, *y*_*j*_) must satisfy Mercer's condition.

## 3. Data Acquisition

**Figure 1:**
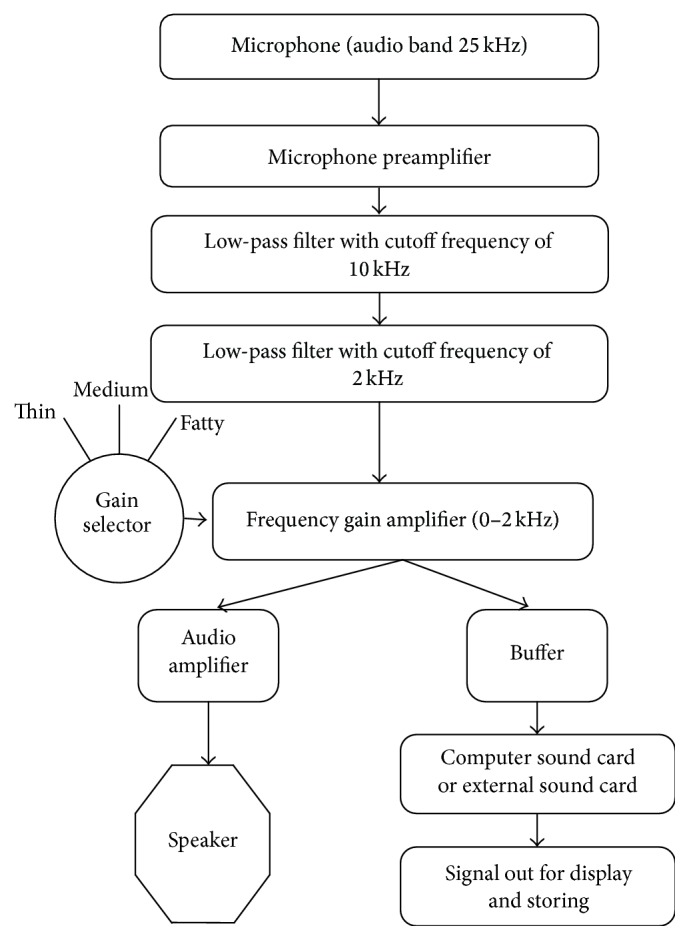
Block diagram of the data acquisition system.

The cardiac sound signals are recorded from normal as well as abnormal male and female subjects using a single channel stethoscope based data acquisition system as shown in Figure 1 and described in [18]. The HS data are recorded from different auscultation location over the body surface of the patients in sitting position and under relaxed conditions (Algorithm 1). The recordings are not associated with any particular age group. The recorded data are arranged in 16-bit PCM, mono audio format and stored as ^*∗*^.wav files at sampling frequency of 8 kHz. The pathological HS are recorded from 16 female and 45 male subjects with various valvular heart diseases. On the other hand, normal HS are collected from 10 female and 15 male subjects. The lung sound records are collected from various resources: Institute of Pulmocare and Research, Kolkata, India, and the Maulana Azad Medical Institute, Delhi, India. The abnormal heart sounds include late systolic murmur, pulmonary stenosis, early systolic murmur, ejection click, aortic insufficiency, and pansystolic murmur.

The whole analysis is implemented on an ACER-PC with 2.39 GHz Intel core 2 quad CPU and 4 GB of RAM. The MATLAB (R2008a, The Mathworks, Inc., Natick, MA) tool is used for conducting all the experiments.

## 4. Methods

**Figure 2:**
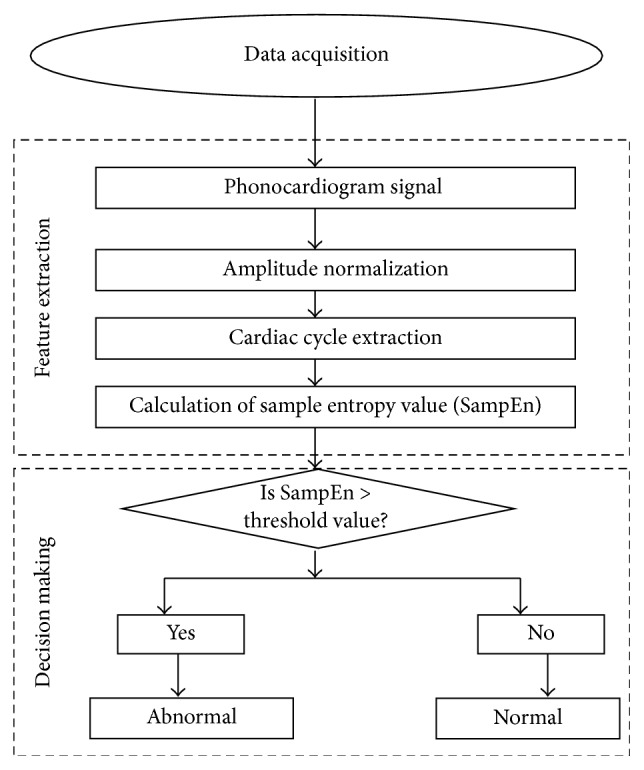
Block diagram of the individual stages involved in cardiac status detection.

The proposed method comprises three stages: data acquisition, feature extraction, and decision making. In the data acquisition stage, heart sounds are recorded from the individual normal and pathological subjects using a stethoscope based system as shown in Figure 1. In the feature extraction stage, robust feature is extracted from the signals by calculating the irregularity parameter of the signals and finally, decision is taken by a machine learning based processing system. The block diagram of the proposed system is depicted in Figure 2.

### 4.1. Amplitude Normalization

The fundamental operation is done to avoid the shortcomings associated with fluctuation in amplitude of the signals for different factors including subject parameters such as age, thickness of the thorax, sex, and also recording condition. The normalized process limits the upper and lower values of amplitude of the signal in a specified range of ±1.

### 4.2. Cardiac Cycle Calculation

A heart sound record contains a series of cardiac cycles. A cardiac cycle consists of four parts: S1, systole, S2, and diastole period. However, the analysis of many cycles at a time increases the complexity and execution time of the data processing system. Moreover, it deteriorates the performance of the classifier system due to the presence of redundant irrelevant information, that is, noise. To resolve this problem a cycle extraction algorithm is required. Researchers have developed various heart sound segmentation algorithms based on auxiliary signal, tempospectral analysis, and envelope representation of the heart sound to localize the primary heart sound components (S1 and S2) [19–24]. In this paper, a new cardiac cycle detection algorithm is presented based on fractal dimension (FD) property of heart sound signal. This executes two operations: firstly, identification of heart sound peaks using Katz algorithm [25] and secondly, calculation of cardiac cycle.

#### 4.2.1. Identification of HS Peaks Using FD

The procedure for identification of HS peaks includes the following steps.

**Step 1 .**

Choose a window of length *M* which is an integer value of 0.05*∗f*_s_, where *f*_s_ is the sampling frequency of the recordings.

**Step 2 .**

Segregate the signal into segment of equal length with a 99% of overlap in order to estimate the FD signal corresponding to HS.

**Step 3 .**

**Figure 3:**
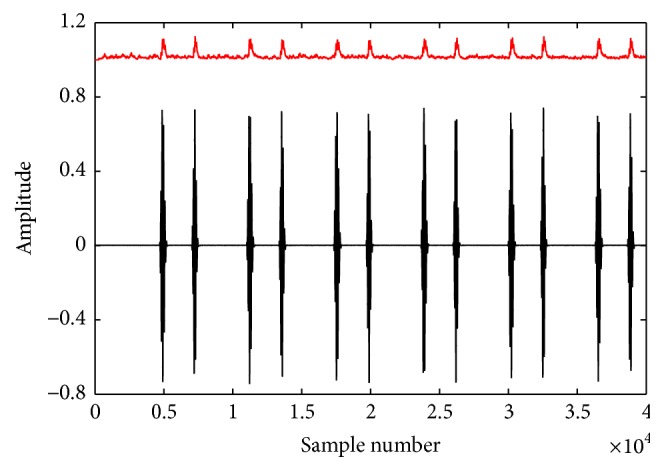
FD signal estimation: waveform of the PCG signal and its corresponding FD signal (upper one).

Calculate FD value for each segment using the Katz algorithm as shown in Figure 3.

#### 4.2.2. Calculation of Cardiac Cycle

**Figure 4:**
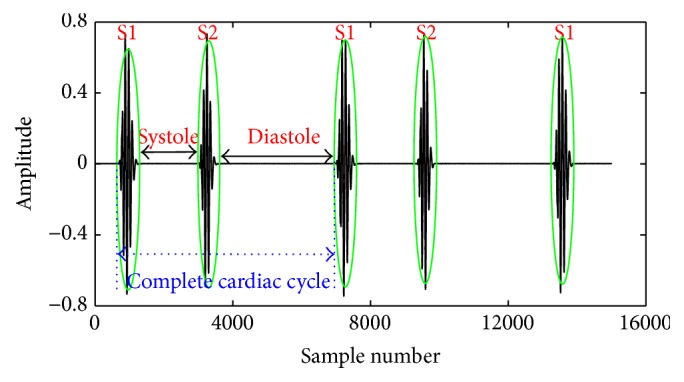
Fundamental components of the cardiac cycle (S1, systole, S2, and diastole).

The cardiac cycle comprises four sequences such as S1 event, systole period, S2 event, and diastole period. Each sequence is bounded by a start and an end point. However, the end point of S1 is merged with the beginning of systole and the end point of later one is also merged with that of S2. Similarly the end point of S2 is merged with the starting point of diastole period. Hence, a cardiac cycle consists of overall five points including three start points and two end points as described in Figure 4. The distance between beginning of S1 and ending of diastole interval gives the cycle duration of the cardiac signal. Let us consider that *d*_s1_ is onset time for S1, *d*_s2_ for S2, *d*_sys_ for systole, and *d*_dia_ for diastole, respectively. Therefore, the duration of the cardiac cycle *C*_*d*_^HS^ is equal to (*d*_s1_ + *d*_sys_ + *d*_s2_ + *d*_dia_).

**Figure 5:**
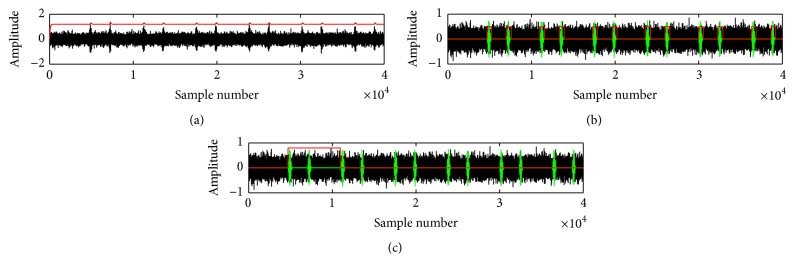
Different steps of cardiac cycle detection: (a) noisy heart sound signal and its corresponding FD signal (above); (b) transition points corresponding to individual events (S1 and S2); (c) estimation of cardiac cycle.

The procedures of cardiac cycle extraction algorithm are provided in Algorithm 2 and the results are shown in Figure 5.

### 4.3. Feature Extraction

This is an important intermediate step of cardiac status detection system. In this stage, the attributes which are useful for modeling the normal and abnormal phonocardiogram signals are extracted. In this study, a statistical parameter called sample entropy is used as feature. The concept of sample entropy was derived from approximate entropy (ApEn) that was first introduced by Pincus. Later on Moorman and Richman modified the approximate entropy by excluding the self-template matching score and defined the new parameter as sample entropy (SampEn) [26]. The sample entropy (*α*) can measure the complexity or irregularity in a signal. This parameter value increases with the irregularity property of the signals and vice versa. The normal heart sound is more regular or less complex in nature than that of the abnormal heart sound signal. Hence, abnormal heart sound gives a higher sample entropy value over the normal one. It is defined as the negative logarithm of an estimate of the conditional probability of the two states that match pointwise for a dimension *m* within a tolerance *r* remain match in dimension *m* + 1. The SampEnt calculation algorithm consists of the several steps that are described next.

**Step 1 .**

The templates of size m are formed from a given time series *s*(*n*), where *n* is the number of data points (here *s*(*n*) represents the heart sound signal) as follows:

(7) Tmj=sj,sj+1,…,sj+m−1,1≦j≦N−m+1.

**Step 2 .**

The distance between vectors *T*_*m*_(*j*) and *T*_*m*_(*i*), that is, *d*[*T*_*m*_(*j*), *T*_*m*_(*i*)] is given by the absolute magnitude of the maximum difference of the corresponding scalar components of these templates and is calculated by

(8) $$\begin{array}{c} d\left[\;T_{m}\left(\;j\;\right),T_{m}\left(\;i\;\right)\;\right]=\underset{k=0,1,\ldots ,m-1}{\max}\left(\;\left|\;s\left(\;j+k\;\right)-s\left(\;i+k\;\right)\;\right|\;\right). \end{array}$$

**Step 3 .**

Imposing the restriction, *d*[*T*_*m*_(*j*), *T*_*m*_(*i*)] ≤ *r*, *i* ≠ *j*, where “*r*” is the threshold that acts as noise filter and *i* ≠ *j*, the number of templates that matches a given vector *T*_*m*_(*i*) is to be detected.

**Step 4 .**

For a signal having *N* ^*m*^(*i*) number of templates matching, the conditional probability for each template is calculated as

(9) $$\begin{array}{c} C^{m}\left(\;r\;\right)=\frac{1}{N-m}\overset{N-m}{\underset{i=1}{\sum }}\frac{N^{m}\left(i\right)}{N-m-1}. \end{array}$$

**Step 5 .**

By changing the value of *m* to *m* + 1 and iterating the above steps we get *C* ^*m*+1^(*r*).

**Step 6 .**

The sample entropy can be computed as negative logarithm of the ratio of the two conditional probabilities as obtained in Steps 5 and 4, which is as follows:

(10) $$\begin{array}{c} \text{SampEnt}\left(\;m,r,N\;\right)=-\ln\left[\;\frac{C^{m+1}\left(r\right)}{C^{m}\left(r\right)}\;\right]. \end{array}$$

The irregularity index or sample entropy value for normal and abnormal heart sounds signals is computed under clean and noisy condition (10 dB to −5 dB). The result is given in Table 1. We can observe from this table that irregularity index value of abnormal heart sound signal is always greater than that of the normal one for both conditions: clean and noisy. As the abnormal HS signal was contaminated with auxiliary murmur signal as a result it produces higher complex pattern over normal signal.

### 4.4. Classification

**Figure 6:**
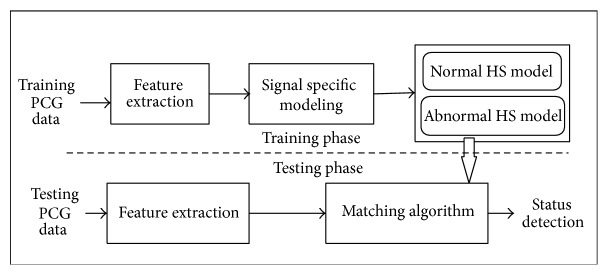
Block diagram of the different stages involved in training and testing process for the assessing of the cardiac status.

This is the final stage of the decision making task regarding the cardiac status. The support vector machine classifier performs the decision making task by executing two functions: training and testing. A suitable structure of the SVM network is formed in the training phase using feature vectors of the known classes. In the subsequent phase, the trained model is employed for recognition of unknown classes. The various stages involved in the classification operation are shown as a block diagram in Figure 6.

## 5. Results

In this study, a composite data set of normal and pathological PCG sounds was used. The decision regarding the cardiac conditions, that is, normal versus abnormal, was made by examining the sample entropy features through a support vector machine classifier. The classifier works through two phases: training and testing. A desired support vector network is constructed by the training process and next this trained network is used to recognize the unknown data. The effectiveness of the proposed method is validated by performing a noise study at a range of SNR (−5 dB to 10 dB). The effectiveness is measured in terms of percentage of classification accuracy (CA%), sensitivity (SEN%), and specificity (SPE%). These measuring units are defined by the following equations:

(11) CA%=TP+TNTN+TP+FN+FP×100,SEN%=TPTP+FN×100,SPE%=TNTN+FP×100.

**Figure 7:**
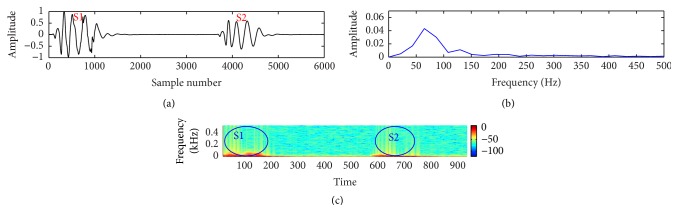
Visual representation of a clean normal signal: (a) waveform of the signal; (b) spectral characteristic of the signal; and (c) spectrogram of the signal.

**Figure 8 fig8:**
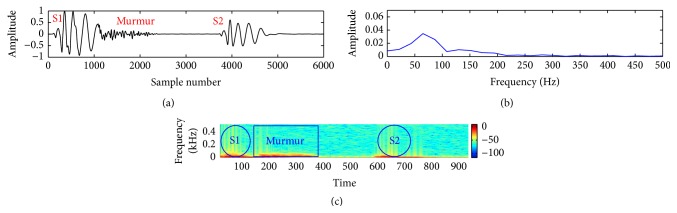
Visual representation of a clean pathological signal (early systolic murmur): (a) waveform of the signal; (b) spectral characteristic of the signal; and (c) spectrogram of the signal.

**Figure 9 fig9:**
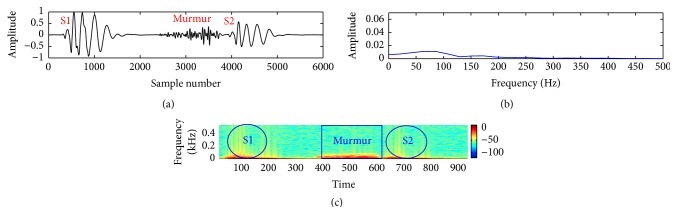
Visual representation of a clean pathological signal (late systolic murmur): (a) waveform of the signal; (b) spectral characteristic of the signal; and (c) spectrogram of the signal.

**Figure 10 fig10:**
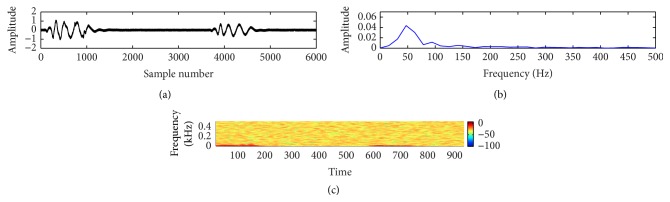
Visual representation of a noisy normal signal: (a) waveform of the signal; (b) spectral characteristic of the signal; and (c) spectrogram of the signal.

**Figure 11 fig11:**
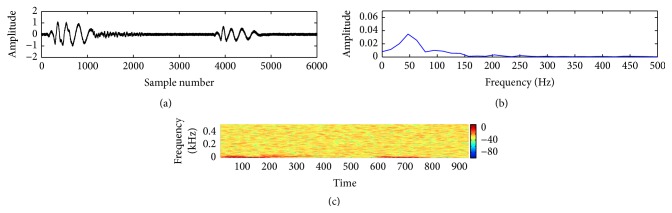
Visual representation of a noisy pathological signal (early systolic murmur): (a) waveform of the signal; (b) spectral characteristic of the signal; and (c) spectrogram of the signal.

**Figure 12:**
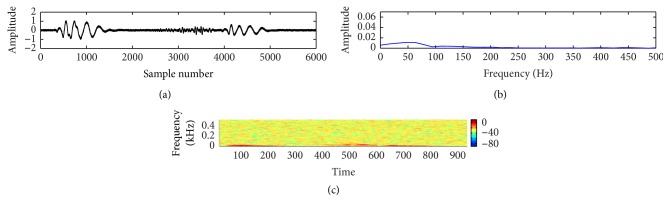
Visual representation of a noisy pathological signal (late systolic murmur): (a) waveform of the signal; (b) spectral characteristic of the signal; and (c) spectrogram of the signal.

The experimental results for clean and noisy database including normal and abnormal subjects are shown in Table 2. The sample entropy based feature extraction technique gives quite better results up to 0 dB SNR. However, visualization tests become unreliable for SNR value lower than 10 dB. It is quite difficult for a doctor to interpret regarding the diseases by listening to and visualizing the noisy recordings of HS. Figures 7 [Fig fig8] [Fig fig9] [Fig fig10] [Fig fig11]–12 show the temporal domain, spectral domain, and spectrogram representations of clean and corrupted HS for both normal and abnormal cases at 10 dB SNR. The abnormal signal data base includes various type of diseases sounds such as early systolic murmur, late systolic murmur, pansystolic murmur, aortic stenosis, pulmonary stenosis, mitral stenosis, and mitral regurgitation. The graphical representations illustrate that external disturbance or noise masks the primary (S1) and secondary (S2) components of cardiac sound signal. The noise contaminated HS signal for normal or different types of abnormal signals produces similar distribution pattern and makes it difficult to distinguish the individual parts of cardiac cycle. The principle of sample entropy feature based classification technique is to categorize the signal into normal and abnormal class based on their irregularity property. The irregularity distribution of the PCG signal is measured by sample entropy. The irregularity distribution signifies the abnormal condition of the heart and regularity distribution highlights the normal condition of the heart. The problem associated with visualization tests may be resolved by the proposed technique because the irregularity property of the signal is less affected by noise. The developed technique performs better for abnormal cases for any SNR levels but its performance degrades for normal cases at high noise level because irregularity distribution of normal signal increases with noise and as a result it shows an abnormal property of signal.

## 6. Conclusion

A new technique based on statistical theorem has been proposed to detect heart status: normal versus abnormal in noisy environment. A decision regarding the different cardiac states is made automatically based on a matching algorithm between known (training) and unknown (test) features variation with the help of a classifier. The developed algorithm can track the structural changes of the signals, if it transits from normal condition to abnormal condition at the initial stage of the diseases. It also removes the difficulties of conventional stethoscope based auscultation tool that makes misinterpretation of the diseases due to corrupted signal. Therefore, the developed technique can be implemented to derive a computer-aided system for enhancing the performance of stethoscope based tool. Furthermore, a detailed analysis is required for a large database of various pathological heart sounds in order to utilize the algorithm for real-time application.

## Figures and Tables

**Algorithm 1:**
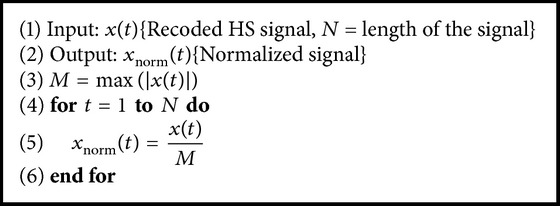
Calculation of normalized signal.

**Algorithm 2:**
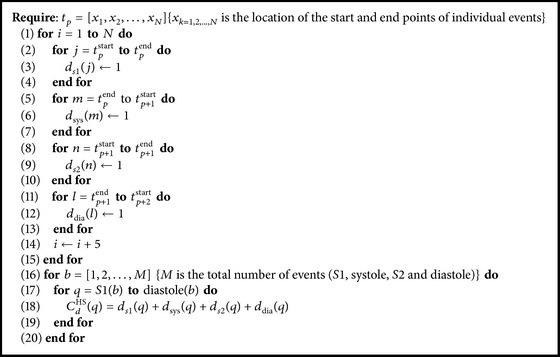
Calculate *C*_*d*_^HS^.

**Table 1:** Irregularity index value for both clean and corrupted database of Gaussian noise.

Pathology	Irregularity index value (*μ* ± *σ*)				
	Clean	10	5	0	−5
Normal	0.15 ± 0.06	0.21 ± 0.04	0.27 ± 0.05	0.41 ± 0.07	0.66 ± 0.17
Abnormal	0.46 ± 0.23	0.70 ± 0.17	0.73 ± 0.16	0.77 ± 0.14	0.86 ± 0.12

*μ*: mean; *σ*: standard deviation.

**Table 2:** Performance of the proposed method for both clean and corrupted database of Gaussian noise.

SNR (dB)	TP	TN	FP	FN	SEN (%)	SPE (%)	ACC (%)
Clean	28	30	0	2	93.33	100	96.67

10	27	28	2	3	90.00	93.32	91.66

5	25	27	3	5	83.33	90.00	88.33

0	24	28	2	6	80.00	93.34	86.68

−5	18	22	8	12	60.00	73.33	66.65

TP: true positive; TN: true negative; FP: false positive; FN: false negative; SEN: sensitivity; SPE: specificity; ACC: accuracy.
